# Exploring the Protective Effects and Mechanism of Huaji Jianpi Decoction against Nonalcoholic Fatty Liver Disease by Network Pharmacology and Experimental Validation

**DOI:** 10.1155/2022/5440347

**Published:** 2022-09-26

**Authors:** Hongkun Xue, Yu Wang, Hongwei Xiang, Qi Song, Guowei Zhang, Jianguo Wang, Shaoqin Ge

**Affiliations:** ^1^College of Traditional Chinese Medicine, Hebei University, No. 342 Yuhua East Road, Lianchi District, Baoding 071002, China; ^2^Hebei University Health Science Center, No. 342 Yuhua East Road, Lianchi District, Baoding 071002, China; ^3^Department of Clinical Lab, Affiliated Hospital of Hebei University, No. 212 Yuhua East Road, Lianchi District, Baoding 071002, China

## Abstract

This paper was designed to predict the mechanisms of the active components of Huaji Jianpi Decoction (HJJPD) against nonalcoholic fatty liver disease (NAFLD) based on network pharmacology-combined animal experiments. The candidate compounds of HJJPD and its relative targets were obtained from TCMSP and PharmMapper web server, and the intersection genes for NAFLD were discerned using OMIM, GeneCards, and DisGeNET. Then, the target protein-protein interaction (PPI) and component-target-pathway networks were constructed. Moreover, gene function annotation (GO) enrichment and Kyoto Encyclopedia of Genes and Genomes (KEGG) pathway analysis were performed to study the potential signaling pathways associated with HJJPD's effect on NAFLD. Molecular docking simulation was preformed to validate the binding affinity between potential core components and key targets. Eventually, the candidate targets, the possible pathway, and the mechanism of HJJPD were predicted by the network pharmacology-based strategy, followed by experimental validation in the NAFLD mice model treated with HJJPD. A total of 55 candidate compounds and 36 corresponding genes were identified from HJJPD that are associated with activity against NAFLD, and then the network of them was constructed. Inflammatory response and lipid metabolism-related signaling pathways were identified as the critical signaling pathways mediating the therapeutic effect of the active bioactive ingredients on NAFLD. Compared with the model group, the liver wet weight, liver/body ratio, the levels of total cholesterol (TC), triglyceride (TG), aspartate aminotransferase (AST), alanine aminotransferase (ALT), and high-density lipoprotein (HDL) in serum in the HJJPD low-dose (17.52 g/kg·d), medium-dose (35.04 g/kg·d), and high-dose (70.07 g/kg·d) groups significantly decreased (*P* < 0.05). Light microscope observation shows that HJJPD could control the degree of lipid denaturation of the mouse liver tissue to a great extent. RT-qPCR results show that the mRNA expression levels of peroxisome proliferative activated receptor gamma (PPARG), tumor necrosis factor-*α* (TNF-*α*), antiserine/threonine protein kinase 1 (AKT1), and prostaglandin-endoperoxide synthase (PTGS2) in the liver tissues of the three HJJPD groups (17.52 g/kg·d, 35.04 g/kg·d, and 70.07 g/kg·d) were significantly lower than those in the model group (*P* < 0.05). HJJPD can exert its effect by inhibiting hepatic steatosis and related mRNA expression and decreasing the levels of other liver-related indexes. This study suggested that HJJPD exerted its effect on NAFLD by modulating multitargets with multicompounds through multipathways. It also demonstrated that the network pharmacology-based approach might provide insights for understanding the interrelationship between complex diseases and interventions of HJJPD.

## 1. Introduction

With the globalization of obesity and relative metabolic disorders, excess nutrition leads to the prevalence of nonalcoholic fatty liver disease (NAFLD) worldwide [1]. There are billions of people who suffer from NAFLD worldwide, and the rapid increasing prevalence of NAFLD is more than 25% in recent years [2]. NAFLD, as a very common disease in overweight and obese people, is characterized by excessive fat deposition in hepatocytes, often accompanied by inflammation and hepatocyte damage. NAFLD is linked to not only an increased risk of liver illness, disability, and death but also an increased risk of metabolic syndrome (MetS), type 2 diabetes, and cancer-related diseases [3]. In addition, NAFLD has become the most frequent chronic liver disease because of the rising prevalence of obesity and MetS, with the leading source of aberrant liver biochemical indicators in health examinations. Nevertheless, the potential molecular mechanism of NAFLD is still unclear. The theories of many researchers have put forward some assumptions. According to the numerous reports, “Two-hit hypothesis” has been widely recognized, which indicates that the accumulation of fat in the liver is easy to induce secondary stress in the liver, including oxidative stress, inflammation, and cytokines [4–6]. However, there are rare reports on the effective therapeutic strategy for NAFLD, except for the appropriate control of exercise and diet. Hence, it is urgent to find the alternative medicine or effective therapy of NAFLD.

Traditional Chinese medicine (TCM) has been widely applied for the regulation of the physiological function of the human body with the advantage of holistic concept and differentiation treatment [7, 8]. Many TCM prescriptions have been proposed to treat the complex metabolic disease of NAFLD, such as PingTang No.5 capsule, Jiangzhi Decoction, Fufang Zhenzhu Tiaozhi capsule, Qushi Huayu Decoction, etc. [9, 10] showed the significant therapeutic effect for antioxidant, anti-inflammation, insulin sensitiz, etc.[11, 12].

Network pharmacology is a thriving interdisciplinary technology that is related to Chinese traditional medicine, chemical informatics, computer science, and bioinformatics, and it is an efficient and powerful tool for studying the bioactive ingredients and mechanism of Chinese traditional medicine. Huaji Jianpi Decoction (HJJPD), as a folk prescription, was derived from the famous TCM prescription of Zhizhu pill, Liujunzi Detection, and Jiaosanxian, composed of *Atractylodes macrocephala* (Baizhu), *Astragalus memeranaceus* (Fisch.) Bge. Var. Mongholicus (Bge.) Hsiao (Huangqi), *Pinellia ternata* (Thunb.) Breit. (Banxia), *Citrus reticulata* Blanco (Chenpi), *Wolfiporia cocos* (F. A. Wolf) Ryvarden & Gilb. (Fuling), *Coix lacryma-jobi* L. var mayuen (ROman.) Stapf (Yiyi Ren), *Atractylodes* Lancea (Thunb.) DC. (Cangzhu), *Citrus aurantium L*. (Zhishi), *Alisma plantago-aquatica* Linn. (Zexie), *Ligusticum chuanxiong* hort (Chuanxiong), and *Nelumbo nucifera* Gaertn (Heye) [13]. As per the reports, Zhizhu pill, Liujunzi decoction, and Jiaosanxian had been used to regulate the dysfunction of the spleen and stomach, and it showed the obvious reliever effect on the fat accumulation of the liver [14]. The main effective components of *G. uralensis, C. pinnatifida,* and *C. reticulata*, as the anti-inflammatory and lipid-lowering drug, have remarkable effect in the treatment of liver diseases and NAFLD [15]. Especially, *C.pinnatifida* was a common therapeutic drug that was added in the TCM prescription for the treatment of NAFLD and liver cirrhosis. Hence, HJJPD may be the superior therapeutic strategy for NAFLD according to TCM experience and scientific research. Presently, the molecular mechanisms underlying the efficacy of HJJPD remain uncertain. It is quite meaningful to further study the chemical composition and pharmacology of HJJPD for the treatment of NAFLD.

HJJPDs are composed of a variety of TCMs, and they are characterized by multiple components, targets, and pathways, which is of great difficulty for studying the chemical composition and mechanism of HJJPDs [13]. Network pharmacology and molecular docking offer an appropriate opportunity to understand the complex mechanisms, and they have been commonly used for the research of Shufeng Jiedu Capsule, Qingzi Zhitong Decoction, Luohua Zizhu Granule, Huanglian Jiedu Decoction, etc. [16, 17]. The aim of this paper is to search the main bioactive ingredients, potential therapeutic targets, and key pathways responsible for HJJPD in the treatment of NAFLD on the basis of network pharmacology and molecular docking. Subsequently, the effect and mechanisms of HJJPD for the treatment of NAFLD are validated by mice experiments and RT-qPCR analysis.

## 2. Materials and Methods

### 2.1. Experimental Animals

80 SPF male C57BL/6N mice aged 3 weeks and weighing 20 ± 2.0 g were purchased from Beijing Vital River Laboratory Animal Technology Co., Ltd. (Animal Certificate No.: SCXK-2019-0010, Beijing, China) and treated in accordance with the Guide for the Care and Use of Laboratory Animals. The experimental site is the third level scientific research laboratory of traditional Chinese medicine pharmacology, College of Traditional Chinese Medicine, Hebei University (No : TCM-09-315). All animal experiments were reviewed and approved by the Animal Care and Use Committee of Renmin Hospital of Hebei University (IACUC-2018043).

### 2.2. Reagents

Trizol reagent kit, total RNA extraction reagent, and DNA marker were purchased from Aidelai Biotechnology Co., Ltd. (Beijing, China). *Atractylodes macrocephala* (Baizhu), *Astragalus memeranaceus* (Fisch.) Bge. Var. Mongholicus (Bge.) Hsiao (Huangqi), *Pinellia ternata* (Thunb.) Breit. (Banxia), *Citrus reticulata* Blanco (Chenpi), *Wolfiporia cocos* (F.A. Wolf) Ryvarden & Gilb. (Fuling), *Coix lacryma-jobi* L.var.mayuen (ROman.) Stapf (Yiyi Ren), *Atractylodes Lancea* (Thunb.) DC. (Cangzhu), *Citrus aurantium* L. (Zhishi), *Alisma plantago-aquatica* Linn. (Zexie), *Ligusticum chuanxiong* hort (Chuanxiong), and *Nelumbo nucifera* Gaertn (Heye) were obtained from Hebei Anguo medicine market (Hubei Province, China) and identified by Professor Keli Chen of Hubei University of Chinese Medicine. All herbals met the requirements of the Chinese Pharmacopoeia (2020 edition), which have been preserved in the College of Traditional Chinese Medicine, Hebei University (No: TCM-09-315).

### 2.3. Sample Preparation

HJJPD consisted of Baizhu (30 g), Huangqi (30 g), Banxia (10 g), Chenpi (10 g), Fuling (30 g), Yiyi Ren (30 g), Cangzhu (15 g), Zhishi (15 g), Zexie (10 g), Chuanxiong (10 g), and Heye (10 g). The above medicinal materials are fully mixed and then made into a powder sample using a plant powder extractor (ZW-100A, Jingxin Industrial Development Co., Ltd, Shanghai, China). The sample was extracted thrice using traditional Chinese medicine extractor (FY-GZJ3L, Feiyue Instrument Co., Ltd, Hangzhou, China) for 1 h. Following extraction, the samples were centrifuged at 5000 g for 15 min at 25°C using a type centrifuge (KH20R-II, Hunan Kaida Scientific Instrument Co., Ltd, Changsha, China) to obtain the extracts, which were concentrated using a vacuum rotary evaporator (DZFY-2L, Xingke Instrument Co., Ltd, Shanghai, China) at 40°C, and the above concentrated extracts were freeze-dried by vacuum freeze dryer (LGJ-12A, Sihuan QIHANG Technology Co., Ltd, Beijing, China), yielding 20.4 g of water extract.

### 2.4. Screening of Bioactive Ingredients of HJJPD and Target Proteins

The active compounds of HJJPD and protein targets of the collected active compounds were obtained from Integrative Pharmacology-based Research Platform of Traditional Chinese Medicine (TCMIP, http://www.gene2newdrug.com/) and Traditional Chinese Medicine System Pharmacology database and Analysis Platform (TCMSP, https://tcmspw.com/tcmsp.php) on the basis of the rule of five (Molecular weight ≤ 500, the number of hydrogen bond donors ≤ 5, the number of hydrogen bond receptors ≤ 10, lipid-water partition coefficient ≤ 5, and rotatable key ≤ 10 using the PharmMapper web server). Meanwhile, the main compounds of herbs were also retained, which has been extensively studied by reported pieces of research [18–20]. In addition, active compounds without protein targets information in TCMIP and TCMSP were predicted by the platform of Swiss ADME (http://www.swissadme.ch/), and target proteins (probability > 0.1) were selected. The target genes for NAFLD were collected from Gene Cards database (https://www.genecards.org/).

### 2.5. Construction of the NAFLD Target Compound Drug and PPI Network

The target genes related to NAFLD and active components of HJJPD were obtained by the online Venn map platform (http://bioinfogp.cnb.csic.es/tools/venny/index.html), and the collected target genes and active components were obtained for subsequent network analysis as visualized using Cytoscape 3.7.2 software. Cytoscape software (V.3.7.2, https://cytoscape.org/) was applied to visualize the relationship between active compounds and diseases of NAFLD. The core active ingredients were identified in the treatment of NAFLD. The candidate genes were imported into the STRING database (https://string.db.org/) for the construction of PPI network, and the visual networks were established by Cytoscape software (V.3.7.2, https://cytoscape.org/). The key genes were selected according to the degree value.

### 2.6. GO and KEGG Enrichment Analyses

The functional annotation of GO and KEGG pathways, including the relevant biological processes (BP), cellular components (CC), molecular functions (MF), and signal pathways of potential anti-NAFLD targets, were performed using the Metascape platform (http://metascape.org/gp/index.html). The bubble map of GO and KEGG enrichment results was established using R Studio. The visualization and integration of enrichment results were carried out using Enrichlot and ggplot2 R package. The above steps were completed by R software 3.6.2.

### 2.7. Molecular Docking

A molecular docking simulation was conducted to assess the binding energy of the core compounds with the key targets. Autodock Vina 1.5.6 software developed by Olson's research group in Scripps Research Institute was adopted to assess molecular docking [16]. Top 10 active compounds and target proteins were selected for molecular docking according to the high degree value. The 3D structures of compounds in ^*∗*^mol format and target protein in ^*∗*^PDB format were obtained from software Chem office. According to *Homo sapiens* and refinement resolution (1.0∼2.0 Å), the target proteins in ^*∗*^PDB format were selected from the RSCB PDB database (https://www.rcsb.org/). Then, the ligands and water molecules in the target protein were removed using software Pymol 2.5, and then it was stored in ^*∗*^pdbqt format. Lastly, Autodock Vina software was used for molecular docking between active compounds and target proteins [16, 17]. The conformations with optimal binding energy were selected for analysis and mapping with Pymol.

### 2.8. Animal Treatment and Sample Collection

The mice in the normal and model groups (10, 70) were fed normal and high-fat diet, respectively. After 10 weeks, 50 obese mice were randomly divided into 5 groups, including the model group, positive control group, and low-, medium-, and high-dose HJJPD-treated groups. The positive control group was given 0.005 g/mL obeticholic acid by gavage for 6 weeks, and normal saline was given to the control group. Low-, medium-, and high-dose HJJPD-treated groups were given 17.52 g/kg·d, 35.04 g/kg·d, and 70.07 g/kg·d of HJJPD, respectively. Meanwhile, the body weight was recorded at 0, 4, 8, 12, 16, 20, and 24 weeks.

All blood samples were collected from the mice orbit after an overnight fast at 24 weeks, and they were centrifuged (3500 rpm for 15 min, Thermo, American) to collect the supernatant for subsequent experiments. Mice were sacrificed and dissected. Then, the liver tissues were collected and measured for the liver index and subsequent histological analysis. The liver index (g/g) was calculated using

(1) $$\begin{array}{c} \text{liver index}=\frac{\text{liver weight}}{\text{body weight}}\times 100\% \end{array}$$

### 2.9. Biochemical Assays

Aspartate aminotransferase (AST), alanine aminotransferase (ALT), the total cholesterol (TC), triglycerides (TG), low-density lipoprotein (LDL), and high-density lipoprotein (HDL) were analyzed by HITACHI 7600 automatic biochemical analyzer (Hitachi Ltd., Japan).

### 2.10. Preparation of Electron Microscope Specimens

The liver tissues were dissected and taken, cut into 1 mm^3^ pieces on ice, fixed in 2.5% glutaraldehyde solution for 2 h, washed with 0.1 M PBS for 3 times, fixed with 1% osmium acid for 1 h, washed with 0.1 M PBS for 3 times, dehydrated with alcohol (30%, 50%, 70%, 85%, 95%, and 100%) and acetone gradient, and subjected to Epon812 penetration. Semithin sections (0.5–2 nm) were performed on the embedded mass of liver tissue, and toluidine blue staining was performed. Morphological changes of liver in mice were observed under the optical microscope.

### 2.11. RT-qPCR Detection

The hepatic expression of NAFLD-related genes (PPARG, TNF-*α*, AKT1, and PTGS2) were analyzed by RT-qPCR. The RNA extraction of the liver tissue was performed according to the operation guidance in the kit instructions. The nucleic acid protein analyzer detects the quality and concentration of total RNA. The A260/A280 ratio is approximately 2.0, and the RNA content is calculated by test kit. 2.0 *μ*g of total RNA was taken and cDNA was synthesized by reverse transcription (primers are shown in Table 1). A real-time fluorescent quantitative PCR reaction system of 20 *μ*L was carried out for 40 cycles. Normalization was performed with *β*-actin as an internal reference, and the results were expressed as relative mRNA levels.

### 2.12. Statistical Analysis

All experiments were repeated at least three times, and the experimental results are shown as the mean ± SD. Student's *t*-test was applied to calculate the differences between the two groups, while one-way analysis of variance (ANOVA) was applied for comparisons among three groups, and post hoc analyses were carried out with the Newman–Keuls multiple comparison test. Statistical significance was considered at *P* < 0.05 (^#^ indicates *P* < 0.05 compared with the normal group, ^*∗*^ indicates *P* < 0.05 compared with the model group, and ^Δ^ indicates *P* < 0.05 compared with the three different dose groups).

## 3. Results

### 3.1. The Bioactive Ingredients and Potential Targets of HJJPD

HJJPD consists of Baizhu (30 g), Huangqi (30 g), Banxia (10 g), Chenpi (10 g), Fuling (30 g), Yiyi Ren (30 g), Cangzhu (15 g), Zhishi (15 g), Zexie (10 g), Chuanxiong (10 g), and Heye (10 g). HJJPD was extracted using traditional Chinese medicine extractor, and the yield of the extract was 17%. NAFLD, as the most common comorbidity, was the attractive research target for TCM. Baizhu, Fuling, and Huangqin had been reported as the potential TCM for improving NAFLD [21]. According to Lipinski's rule of five and extensive research, a total of 96 compounds were selected as the potential bioactive ingredients from TCMIP database (Table 2), containing 7 types in Bai Zhu, 12 types in Huang Qi, 6 types in Ban Xia, 8 types in Chen Pi, 6 types in Fu Ling, 12 types in Cang Zhu, 9 types in Zhi Shi, 13 types in Ze Xie, 11 types in Chuan Xiong, 6 types in He Ye, and 2 types in Yiyi Ren. The bioactive ingredients were abundant and diverse, including poricoic acid *b*, poricoic acid *a*, alisol *a* monoacetate, kumatakenin, baicalin, etc. Table S1 shows the material basis of HJJPD. 4 compounds were overlapped across different herbs, including (+)-Eudesma-4 (15), 7 (11)-Dien-8-One in Baizhu and Cangzhu, 3-O-trans ferulylquinic acid in Chenpi and Chuanxiong, and Nobiletin, Tangeretin in Chenpi and Fuling. The top 10 compounds by the degree value (the number of times calculated the interaction between bioactive compounds, and potential targets appeared in the NAFLD target compound drug network) with chemical structures, molecular weight (Mw), the number of hydrogen bond donors, hydrogen bond acceptors, rotatable bonds, and lipid-water partition coefficient are as shown in Table 3. Meanwhile, a total of 242 potential targets of these compounds were collected from the TCMIP database, including TNF, IL6, AKT1, IL1B, PPARG, PTGS2, ESR1, LPL, PPARA, and HMGCR.

### 3.2. Construction of Ingredient-Target-Disease and PPI Network

**Figure 1:**
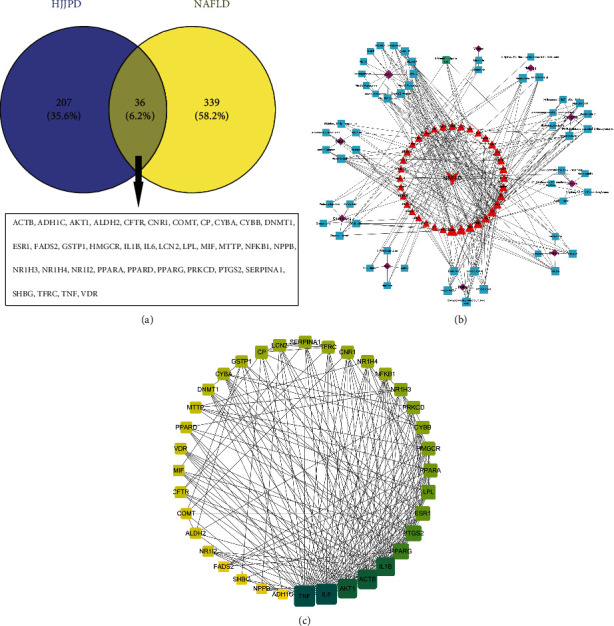
Venn diagram of the targets of HJJPD (a), ingredient-target-disease network (b), and PPI network for the treatment of NAFLD (c).

NAFLD was searched as the key word in the Genecard database [22]. Consequently, 375 targets with relevance score >20 were screened out.  Figure 1(a) shows that a total of 36 target genes were identified by Venn diagram analysis, which matched with 55 active ingredients (Table S2). For elucidating the interaction between active ingredients and potential targets, as well as NAFLD, an ingredient-target-disease network was constructed, including 102 nodes and 307 edges (Figure 1(b)). A PPI network was established to identify the core proteins of HJJPD in the treatment of NAFLD. There were 36 nodes and 195 edges after deleting targets without a relationship linked to other targets. According to the degree value calculated by Cytoscape software, PPARA, LPL, ESR1, PTGS2, PPARG, IL1B, AKT1, ACTB, TNF, and IL6 were the top 10 targets (Figure 1(c)) that were closely related to inflammatory responses, the regulation of lipid metabolism, diabetes, and the addition of melittin in NAFLD.

### 3.3. Functional Enrichment Analysis

**Figure 2:**
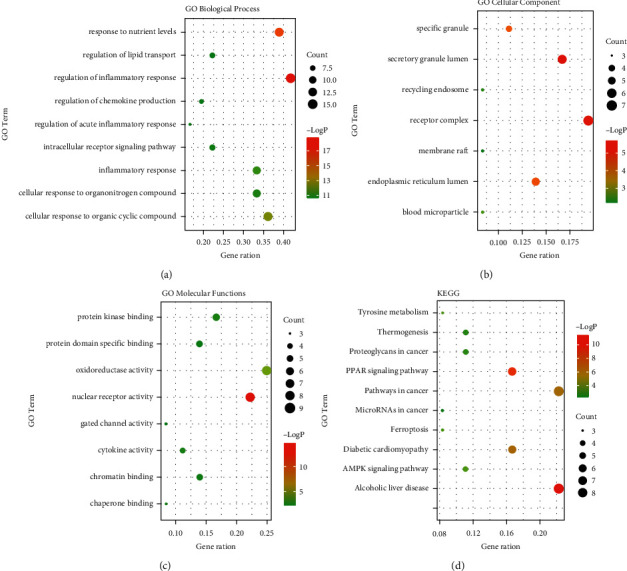
Bubble charts of GO and KEGG enrichment analysis. Note: (a) GO-BP analysis, (b) GO-CC analysis, (c) GO-MF analysis, and (d) KEGG pathways analysis.

The GO functional and KEGG enrichment analyses were performed to study the crucial biological processes of HJJPD in the treatment of NAFLD. As per the GO analysis results, 36 genes were enriched in 508 GO entries that consisted of 2401 biological progress (BP), 9 cellular components (CC), and 25 molecular functions (MF) (*P* < 0.05).  Figure 2 shows the bubble plots and important entries of BP, CC, MF, and KEGG. The BP analysis shows that the potential targets mainly focused on the response to nutritional levels, the regulation of lipid transport, cell response to organic cyclic compounds, organic nitrogen compounds, inflammatory response (Figure 2(a)), etc. The CC analysis suggests that the related targets were mainly concentrated in the receptor complex, secretory granule cavity, endoplasmic reticulum lumen (Figure 2(b)), etc. Moreover, the MF analysis shows that potential targets were primarily focused on oxidoreductase activity, nuclear receptor activity, protein kinase binding (Figure 2(c)), etc. The KEGG enrichment analysis was used to study representative signal pathways related to key targets. 80 signal pathways were obtained (*P* < 0.05).  Figure 2(d) shows the top 10 significantly enriched signal pathways closely related to NAFLD, including the alcoholic liver disease, cancer pathway, PPAR signal pathway, diabetes cardiomyopathy, etc.

### 3.4. Interaction between Ten Protein Targets and Diosgenin

**Figure 3:**
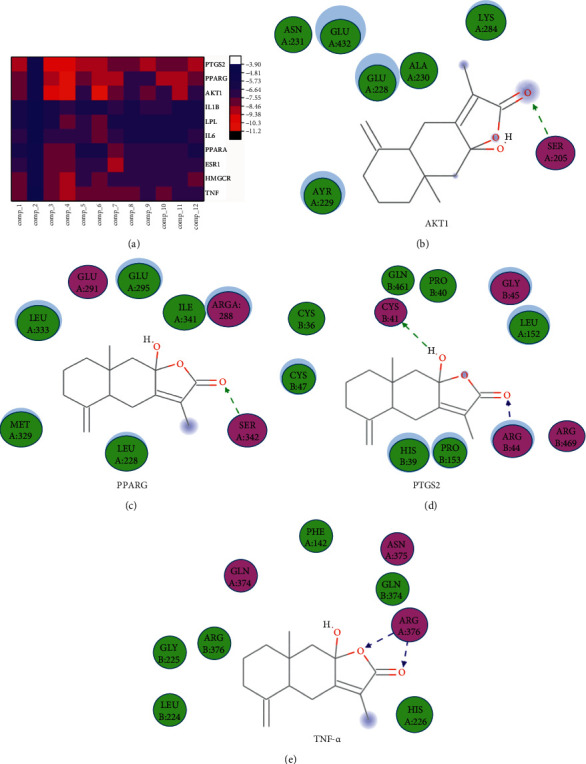
The heat map of the close combination of Atracillin III with various targets, (a) and the three-dimensional map of the docking of Atracillin III with AKT1 (b), PPARG (c), PTGS2 (d), and TNF-*α* (e).

In PPI network analysis, the target of top 10 proteins was docked with the active components of HJJPD, including TNF, IL6, AKT1, IL1*β* (Table S3), PPARG, PTGS2, ESR1, LPL, PPARA, and HMGCR. These targets play an important role in the PPI network and KEGG signaling pathway, and they have a significant impact on glucose and lipid metabolism disorders and inflammation (such as TNF-*α*, AKT1, PPARG, and PTGS2). Binding energy ≤ −5.0 kJ/mol was used as the basis for screening hypoglycemic activity. The results show that the binding energy of all active ingredients to protein was less than −5 kJ/mol, which further indicated that the active ingredients had strong binding activity to protein, expect Poricoic acide A [23]. According to the heat map, Atracillin III could closely bind to the various targets, such as AKT1, PPARG, PTGS2, and TNF-*α*, and the highest binding affinity (-11.2 kJ/mol) was observed in Atractylol III and AKT1 (Figure 3(a)). Figures 3(b)–3(e) show a three-dimensional view of the docking mode of atractylode III with the targets AKT1, PPARG, PTGS2, and TNF-*α*, which mainly depended on the hydrogen bond interaction with Ser205, Ser342, Arg44, Cys41, and Arg52 residues, respectively.

### 3.5. Effects of HJJPD on Body Weight, Liver Weight, and Liver/Body Weight Ratio

**Figure 4:**
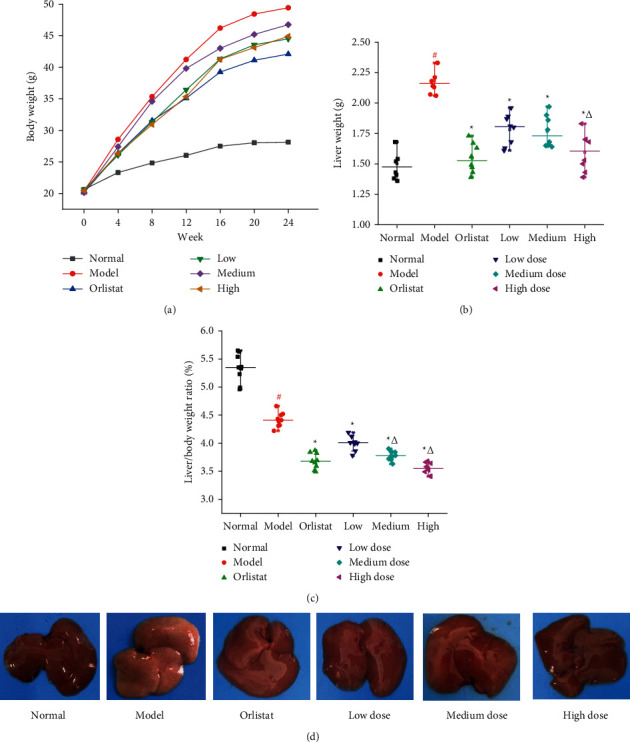
Effects of HJJPD on body weight (a), liver weight (b), liver/body weight ratio (c), and liver morphology (d). Note: values are expressed as mean ± SD, *n* = 10. ^#^*P* < 0.05 compared with the normal group. ^*∗*^*P* < 0.05 compared with the model group. ^Δ^ indicates *P* < 0.05 compared with the three different dose groups.

Obesity is an important risk factor for NAFLD, and the effects of HJPD on mice body and liver weight are shown in Figure 4. The body weight of mice in blank control (normal), model, positive control, and low-, medium-, and high-doses of HJJPD groups increased gradually within a week (Figure 4(a), Table S4). Compared with the normal group, the liver weight of the model group was significantly higher than that of the normal group (Figure 4(b), Table S4), suggesting that high-fat induction increases liver weight. The liver weight of mice in the three dose groups and the positive control group was markedly lower than that in the model group (*P* < 0.05), indicating that HJJPD can inhibit the increase of liver weight to some extent [13]. The liver/body weight ratio of the model group was dramatically lower than that of the normal group (Figure 4(c), *P* < 0.05). In addition, the liver/body weight ratio of mice in the HJJPD treatment group was significantly lower than that in the model group (Figure 4(c), *P* < 0.05).  Figure 4(d) shows that the liver of the normal control group has a ruddy color, a smooth surface, a sharp edge, a complete capsule, and a soft texture after 10 weeks of modeling. The liver of the model group was dark yellowish brown, swollen, with passive edges, dense coating, and evenly distributed particles on the surface, suggesting that liver fat steatosis occurred. HJJPD treatment group and positive control group were carnation. The results show that HJJPD can inhibit liver steatosis [24].

### 3.6. Effects of HJJPD on Blood Lipid and Liver Function

**Figure 5:**
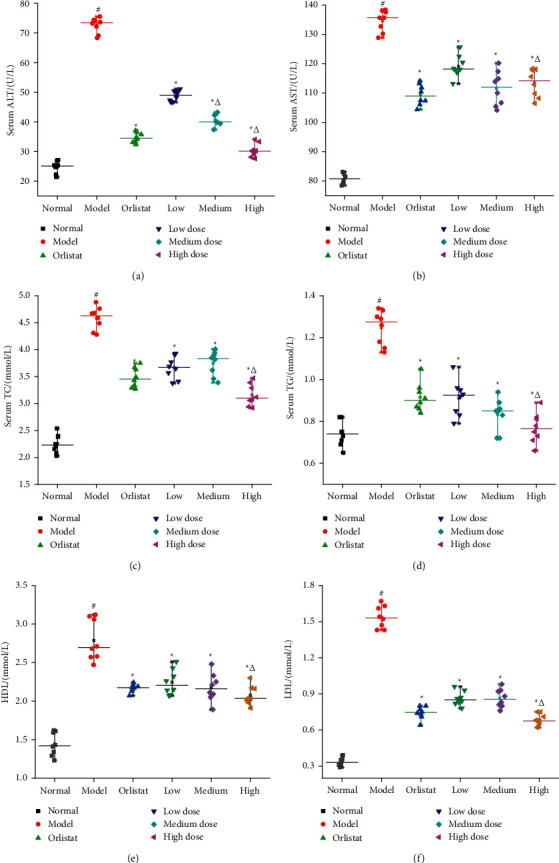
Effects of HJJPD on the levels of ALT (a), AST (b), TC (c), TG (d) in serum, HDL (e), and LDL (f). Note: values are expressed as mean ± SD, *n* = 10. ^#^*P* < 0.05 compared with the normal group. ^*∗*^*P* < 0.05 compared with the model group. ^Δ^ indicates *P* < 0.05 compared with the three different dose groups.

Blood biochemical index can provide the information of various ions, sugars, lipids, proteins, enzymes, hormones, and various metabolites, which is conductive to the determination of treatment effect.  Figure 5 shows the effects of HJJPD on the levels of ALT, AST, TC, and TG in serum, HDL, and LDL. Compared with the normal control group, the levels of ALT, AST, TC, and TG in serum, HDL, and LDL in the model group were significantly higher, implying that high fat could induce liver injury and liver inflammation in mice (Table S4). These results were consistent with the liver morphology. Compared with the model control group, the levels of ALT, AST, TC, and TG in serum, HDL, and LDL in the HJJPD treated groups and the positive control group were significantly lower than those in the model group. The results show that HJJPD can protect the liver from injury [13].

### 3.7. Effect of HJJPD on Liver Histopathology

**Figure 6:**
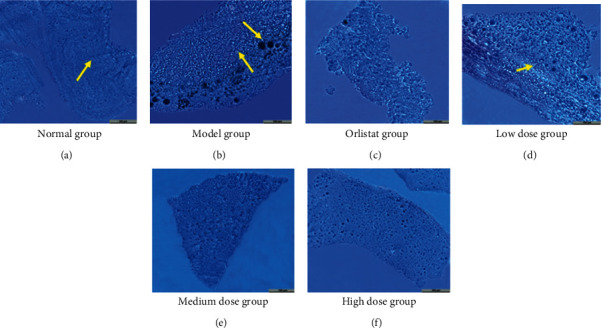
Morphological observation of the liver tissue (×200) in each group of mice. (a) Normal group, (b) model group, (c) orlistat group, (d) low-dose group, (e) medium-dose group, and (f) high-dose group.

Figure 6 shows the liver histopathological changes of the model group, HJJPD-treated groups, and the positive control group.  Figure 6(a) shows that the semithin sections of the liver of the normal group showed a normal lobular structure with a well-structured hepatocyte.  Figure 6(b) observes that the model group showed a large number of lipid droplet vacuoles in the liver tissue, suggesting that liver cells are destroyed and fat droplets are accumulated in large amounts, and the liver has undergone steatosis [25]. Compared with the model group, a small number of fat vacuoles were observed in the liver of the low dose group, and the morphology of the liver cells was relatively good (Figure 6(d), Table S5). There was no or relatively less accumulation of fat droplets in the liver tissue of the orlistat and medium-dose and high-dose groups, which significantly reduced hepatic steatosis, and the hepatocyte boundaries were relatively clear (Table S5). These results suggested that HJJPD has protective effect on NAFLD.

### 3.8. Effect of HJJPD on the mRNA Expression Levels of NAFLD-Related Genes

**Figure 7:**
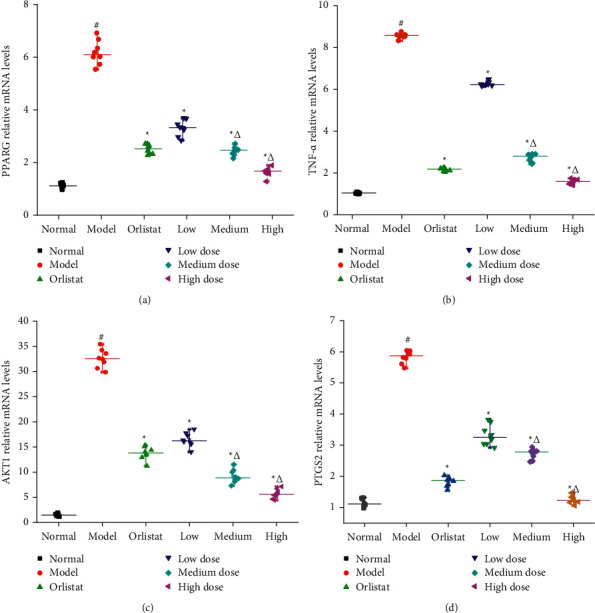
Effect of HJJPD on the mRNA expression levels of NAFLD-related genes. Note: values are expressed as mean ± SD, *n* = 10. ^#^*P* < 0.05 compared with the normal group. ^*∗*^*P* < 0.05 compared with the model group. ^Δ^indicates *P* < 0.05 compared with three different dose groups.

To explore the effect of HJJPD on NAFLD, relevant signaling pathways (TNF-*α*, AKT1, PPARG, and PTGS2) predicted by network pharmacology were estimated by RT-qPCR (Figure 7). In the normal group, the mRNA expression levels of PPARG, TNF-*α*, AKT1, and PTGS2 were lower than those in the model group (Figure 7, *P* < 0.05). Compared with the model group, the mRNA expression levels of PPARG, TNF-*α*, AKT1, and PTGS2 in the HJJPD-treated groups and the positive control group were significantly lower than those in the model group (*P* < 0.05). These results suggest that HJJPD has protective effects on NAFLD by inhibiting inflammatory factors, glucose, and lipid metabolism.

## 4. Discussion

Although several decades of NAFLD research has made significant scientific progress in the pathogenesis and therapeutic target, effective drugs are currently under clinical development [26]. NAFLD is a clinicopathological syndrome caused by excluding alcohol and other liver damaging factors. Its main characteristics are steatosis and fat storage in liver parenchyma cells [27]. Many researches have indicated that the occurrence and development of nonalcoholic fatty liver are closely related to high-fat diet, lipid metabolism disorder, obesity, hypertension, arteriosclerosis, oxidative stress, and other factors [28, 29]. The pathogenesis of NAFLD is not completely clear so far. However, it has been proved that insulin resistance, oxidative stress response, lipid peroxidation damage, endoplasmic reticulum stress, and intestinal flora imbalance are closely related to the occurrence and development of NAFLD [30]. Currently, there are no approved effective therapeutic medicine for NAFLD. TCMFs exhibits obvious glucose and lipid metabolism, anti-inflammatory properties, antioxidant stress, antifibrosis, and gut microbiota modulation properties, which have been extensively studied for the treatment of NAFLD [31]. Meanwhile, the Chinese patent drug composed of Chinese medicinal herbs was applied to improve NAFLD in clinical treatment with the unique holistic concept and differentiation treatment advantages, such as Huazhirougan granule, Dang Fei Li Gan Capulse, Shen Ze Shu Gan Caspulse, San Qi Zhi Gan Pills, Bai zhu, Ze Xie, Fu Ling, Huang Qi, etc. [13]. It was a good candidate for medicine because of its ability to significantly improve glucose and lipid metabolism and inflammatory response in NAFLD. However, Chinese medicinal herbs have a variety of flavonoids, terpenoids, organic acids, steroids, and tannins with a comprehensive biological effect. Meanwhile, TCM prescriptions emphasize on the adjustment of individualized therapy based on syndrome differentiation. According to the characteristics of spleen deficiency and dampness obstruction syndrome, HJJPD was established from the famous TCM prescription of Zhizhu pill, Liujunzi Detection, and Jiaosanxian, which consisted of Baizhu (30 g), Huangqi (30 g), Banxia (10 g), Chenpi (10 g), Fuling (30 g), Yiyi Ren (30 g), Cangzhu (15 g), Zhishi (15 g), Zexie (10 g), Chuanxiong (10 g), and Heye (10 g) [32]. In addition, many studies have also confirmed the good therapeutic effect of HJJPD on NAFLD [33]. However, the multicomponent and multitarget characteristics of HJJPD have become the major obstruction to establish the molecular mechanisms underlying their pharmacological activity.

Network pharmacology is a thriving interdisciplinary science and technology that is related to Chinese traditional medicine, chemical informatics, computer science, and bioinformatics, which was an efficient and powerful tool for studying the bioactive ingredients and mechanism of Chinese traditional medicine [34–36]. The candidate components and targets of HJJPD were collected from TCMIP, TCMSP, and SwissADME, respectively, and they were analyzed by Lipinski's rule of five and network pharmacology. As per results, 55 candidate compounds and 36 corresponding genes were identified, and they were associated with the HJJPD activity against NAFLD. Top 10 active ingredients of HJJPD for improving NAFLD were screened using the degree value, such as poricoic acid *b*, poricoic acid *a*, alisol *a* monoacetate, kumatakenin, baicalin, etc. Poricoic Acid A∼D were the main bioactive components of Fu Ling extract, which could ameliorate and treat hepatic steatosis based on the regulation of lipid metabolism, inhibition of oxidative stress, and activation of autophagy depended on the AMPK pathway. Alisol A 24-acetate is an effective component of Ze xie extract. Many researches have confirmed that Alisol A 24-acetate could obviously alleviate lipid deposition in the liver cell by promoting ABCG1 and ABCA1 expression at the mRNA and protein levels. Baicalin, as the good prospect in the treatment of NAFLD, is an important component of Ban xia. Baicalin can partly reduce liver lipid accumulation by regulating the expressions of SREBP-1c, FAS, PPAR*α*, and CPT1a. Some pieces of research also have revealed that baicalin could regulate the metabolism of free fatty acids to improve NAFLD by activating AMPK/SREBP signaling pathway [37, 38]. These results show the abundant and diverse material basis of HJJPD in the treatment of NAFLD. According to PPI network analyses, TNF, IL6, AKT1, ACTB, IL1B, PPARG, PPARA, LPL, ESR1, and PTGS2 were the top 10 targets for HJJPD to treat NAFLD. Previous reports have shown that these targets were closely related to the regulation of inflammatory response, lipid metabolismdiabetes mellitus, and oxidative stress [39, 40]. For example, TNF and IL6 are the promoters of inflammatory responses, which play a major role in many inflammatory cytokines and accelerate NAFLD progression [41]. PPARG is mainly responsible for lipid catabolism, and it is related to NAFLD, obesity, diabetes, arteriosclerosis, etc. [42].

In PPI network analysis, the target of top 10 proteins is docked with the active components of HJJPD, including TNF, IL6, AKT1, IL1*β*, PPARG, PTGS2, ESR1, LPL, PPARA, and HMGCR. These targets play an important role in the PPI network and KEGG signaling pathway, and they have a significant impact on glucose, lipid metabolism disorders, and inflammation (such as TNF-*α*, AKT1, PPARG, and PTGS2). Molecular docking is an important method to realize structure-based drug design and screening by studying the interaction between ligand molecules and receptor molecules, predicting their affinity and binding mode [16]. The principle of molecular docking is that the binding of ligand (PDB format of protein) and receptor (small molecule active ingredients) must meet the principle of mutual matching, i.e., the geometry, electrostatic, hydrogen bond, and hydrophobic interaction of ligand and receptor are complementary and matched [17]. In this study, binding energy ≤ −5.0 kJ/mol was used as the basis for screening hypoglycemic activity. The results show that the binding energy of all active ingredients to protein was less than −5 kJ/mol, which further indicated that the active ingredients had strong binding activity to protein, expect Poricoic acide A [23]. According to the heat map, Atracillin III could closely bind to the various targets, such as AKT1, PPARG, PTGS2, and TNF-*α*, and the highest binding affinity (−11.2 kJ/mol) was observed in Atractylol III and AKT1 (Figure 3(a)). Figures 3(b)–3(e) show a three-dimensional view of the docking mode of atractylode III with the target AKT1, PPARG, PTGS2, and TNF-*α*, which mainly depended on the hydrogen bond interaction with Ser205, Ser342, Arg44, Cys41, and Arg52 residues, respectively.

These possible key components and targets play a vital role for HJJPD in the alleviation of NAFLD. There is good binding activity between the top 10 components (Poricoic Acid A∼B, Atractylenolide III, Baicalin, etc.) and top 10 target proteins (TNF, IL6, AKT1, ACTB, IL1B, PPARG, PPARA, LPL, ESR1, PTGS2). These results are consistent with those reported by Wang et al. [32] and Zhu et al. [33]. The binding energies of top 10 active ingredients to top 10 protein targets were less than −5 kJ/mol, which further indicated that the active ingredients had strong binding activity to protein, except Poricoic acid A. The possible signaling pathways of HJJPD in the treatment of NAFLD were further analyzed by the GO functional and KEGG enrichment analyses. As per results, HJJPD predominantly regulated the nutrient levels, lipid transport, and inflammatory response by participating in the pathway in alcoholic liver disease, cancer, PPAR and MAPK signaling pathways, etc. For example, PPARs bind to ligands, and activate the heterodimerization of retinol X receptor (RXR). Nuclear receptor coactivator cooperates with PPAR-RXR to supplement and stabilize the active transcription complex, which can regulate lipid metabolism, fat formation, and inflammatory gene expression [43]. MAPK signal transduction is closely related to the regulation of adipocyte differentiation, which is a key pathway for the treatment of NAFLD [44]. These results adequately supported the unique characteristics and advantage of multiple targets and multiple effect of TCM in the NAFLD treatment.

Subsequently, NAFLD mice induced by HDF are applied to further ascertain the function of HJJPD in the amelioration of NAFLD by the validation experiment. When the synthesis and secretion of TC in hepatocytes are damaged and cannot be balanced, lipids rapidly deposit in hepatocytes and cannot be excreted, leading to hepatocyte disease and nonalcoholic fatty liver [45]. A large number of studies have indicated that the levels of TG, LDL, and TC in patients with moderate and severe nonalcoholic fatty liver significantly increased, whereas the level of HDL decreased, which indicated that patients with nonalcoholic fatty liver had lipid metabolism disorders, and excessive fat deposition was positively correlated with the severity of nonalcoholic fatty liver [46, 47]. In addition, ALT and AST can reflect the damage of hepatocytes. In case of the steatosis of hepatocytes, the disorder of lipid metabolism, fatty acid damage to hepatocytes, and high transaminase, the levels of ALT and AST in patients with nonalcoholic fatty liver increase significantly [48]. The experiment results show that HJJPD (17.52 g/kg·d, 35.04 g/kg·d, and 70.07 g/kg·d) could significantly decrease the levels of TC, TG ALT, AST, HDL, and LDL (Figure 5). These results are consistent with those reported by Wang et al., [32]. Moreover, HJJPD (17.52 g/kg·d, 35.04 g/kg·d, and 70.07 g/kg·d) could obviously decrease the levels of liver weight and liver/body weight ratio (Figure 4). These results indicate that HJJPD can prevent and treat NAFLD to some extent. Pathological biopsy was the “golden standard” for judging the diagnosis of NAFLD by pathological diagnostic criteria. The experiment results of toluidine blue stained liver reveal that HJJPD could significantly reduce hepatic steatosis in a dose-dependent manner (Figure 6). These results are consistent with those reported by Zhu et al. [33]. Compared with the model group, the hepatocyte boundaries treated by HJJPD were relatively clear, and the accumulation of fat droplets in the liver tissue was absent or relatively less. RT-qPCR was performed to test the mRNA expression levels of PPARG, TNF-*α*, AKT1, and PTGS2 for validation purposes. The experiment results show that HJJPD (17.52 g/kg·d, 35.04 g/kg·d, and 70.07 g/kg·d) could obviously inhibit the mRNA expression levels of PPARG, TNF-*α*, AKT1, and PTGS2 in the liver and adipose tissues, indicating that HJJPD can regulate the inflammatory response and lipid metabolism and reduce the role of hepatic steatosis [13]. These experiment results verified the prediction information of molecular mechanism using network pharmacology and molecular docking and demonstrated the significant therapeutic effect and research value of HJJPD on NAFLD. In conclusion, HJJPD has the potential to treat NAFLD.

## 5. Conclusion

Taken together, the present study puts forward for the first time a comprehensive strategy that combines pharmacological experiments and network pharmacology methods to explore the material basis of HJJPD and its possible mechanisms of the active components of HJJPD against NAFLD. Through active ingredient screening, target prediction, PPI network construction, KEGG pathway, GO biological process analysis, and experimental verification, HJJPD protective mechanism for NAFLD was clarified. It has been demonstrated that inflammatory response- and lipid metabolism- related signaling pathways were identified as the critical signaling pathways mediating the therapeutic effect of the active bioactive ingredients on NAFLD. HJJPD (17.52 g/kg·d, 35.04 g/kg·d, and 70.07 g/kg·d) could significantly decrease the liver wet weight, liver/body ratio, and the levels of TC, TG, AST, ALT, and HDL in serum, and reduce the mRNA expression levels of PPARG, TNF-*α*, AKT1, and PTGS2 in the liver tissues. All in all, HJJPD could ameliorate HFD-induced NAFLD by regulating the inflammatory response and lipid metabolism and decreasing levels of liver-related indexes and the role of hepatic steatosis. This study provides a scientific basis for the further analysis of the clinical application of CCT in NAFLD. However, the further downstream mechanism of HJJPD treatment still needs to be investigated. Additionally, to develop more inexpensive and safer clinical applications, we need to analyze the active components of HJJPD and explore how HJJPD treats NAFLD using different signaling pathways related to inflammation and lipid metabolism.

## Figures and Tables

**Table 1:** Prime sequence of targeted genes and *β*-actin.

Gene	Direction	Sequence
*β*-Actin	Forward	ACTCATCGTACTCCTGCTTGCTGA
	Reverse	AGGGAAATCGTGGGTGACATCAAA

TNF-*α*	Forward	CAGATTGACCTCAGCGCTGAGTTG
	Reverse	ACCCTCACACTCAGATCATCTTCT

PPARG	Forward	CTGGCCTCCCTGATG AATAA
	Reverse	GGCGGTCTCCACTGAGAATA

AKT1	Forward	TGCAGTGGACCACAGTCATT
	Reverse	GGGACACCTCCATCTCTTCA

PTGS2	Forward	TGCACTATGGTTACAAAAGCTGG
	Reverse	TCAGGAAGCTCCTTATTTCCCTT

**Table 2:** Bioactive ingredients of HJJPD in NAFLD treatment.

Herbal	Abbreviation	Number of compounds	Common compounds
*Atractylodes macrocephala*	Baizhu	7	(+)-Eudesma-4(15), 7(11)-Dien-8-One
*Astragalus memeranaceus* (Fisch) Bge. Var. mongholicus (Bge) Hsiao	Huangqi	12	No
*Pinellia ternata* (Thunb) Breit.	Banxia	6	No
*Citrus reticulata* Blanco	Chenpi	8	Nobiletin Tangeretin 3-O-trans ferulylquinic acid
*Wolfiporia cocos* (F.A. Wolf) Ryvarden & Gilb.	Fuling	6	Nobiletin Tangeretin
*Coix lacryma-jobi* L.var.*mayuen* (Roman.) Stapf	Yiyi Ren	2	No
*Atractylodes Lancea* (Thunb.) DC.	Cangzhu	12	(+)-Eudesma-4(15), 7(11)-Dien-8-One
*Citrus aurantium L.*	Zhishi	9	No
*Alisma plantago-aquatica Linn*.	Zexie	13	No
*Ligusticum chuanxiong* Hort	Chuanxiong	11	3-O-trans Ferulylquinic acid
*Nelumbo nucifera* Gaertn	Heye	2	No

**Table 3:** Top 10 Bioactive ingredients of HJJPD in NAFLD treatment.

Molecule ID	Molecule name	Molecular formula	Structure	The rule of five				
				MW	H Donors	H acceptors	Log P	Rotatable bonds
Compound 1	Poricoic acid B	C_30_H_44_O_5_	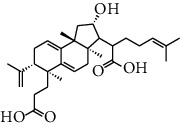	484.66736	3	5	5.688	9

Compound 2	Poricoic acid A	C_31_H_46_O_5_	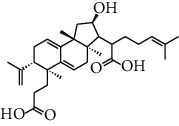	498.69394	3	5	5.994	10

Compound 3	25-Hydroxy-3-epidehydrotumulosic Acid	C_31_H_48_O_5_	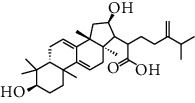	500.70982	4	5	4.528	6

Compound 4	Atractylenolide III	C_15_H_20_O_3_	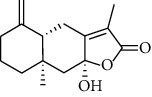	248.31749	1	3	2.924	0

Compound 5	Alisol A monoacetate	C_32_H_52_O_6_	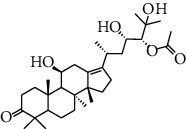	532.75167	3	7	4.395	7

Compound 6	Alisol E 24-acetate	C_32_H_52_O_6_	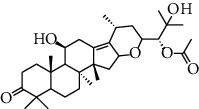	532.75167	3	6	4.395	7

Compound 7	Alisol E 23-acetate	C_32_H_52_O_6_	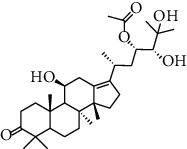	532.75167	3	6	4.395	7

Compound 8	Kumatakenin	C_17_H_14_O_6_	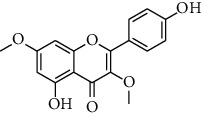	314.28946	2	6	2.323	3

Compound 9	Poricoic acid D	C_31_H_46_O_6_	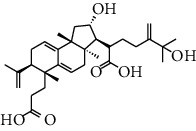	514.69334	4	6	4.777	10

Compound 10	Lauric acid	C_12_H_24_O_2_	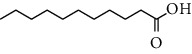	200.31775	1	2	4.568	10

Compound 11	Epigallocatechin	C_15_H14O_6_	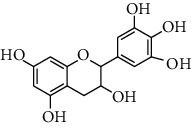	290.26806	5	6	2.021	1

Compound 12	Baicalin	C_21_H_18_O_11_	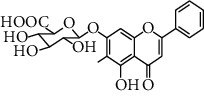	446.36102	3	6	0.608	11

## Data Availability

The data used to support the findings of this study are included within the supplementary information files.

## Supplementary Materials

Supplementary Materials Table S1: the active ingredients of HJJPD. Table S2: the active ingredients of HJJPD in NAFLD. Table S3: the target of top 10 proteins. Table S4 and Table S5: the raw data of manuscript drawing.
