# Supplementary material for: Exploring the Protective Effects and Mechanism of Huaji Jianpi Decoction against Nonalcoholic Fatty Liver Disease by Network Pharmacology and Experimental Validation
Source: Evid Based Complement Alternat Med. 2022 Sep 26;2022:5440347. doi: 10.1155/2022/5440347 (PMC9529445; doi:10.1155/2022/5440347)
Supplement: Supplementary Materials — Table S1: the active ingredients of HJJPD. Table S2: the active ingredients of HJJPD in NAFLD. Table S3: the target of top 10 proteins. Table S4 and Table S5: the raw data of manuscript drawing. [file 5440347.f1.doc]

**Table of Contents**

| **Contents** | page |
| --- | --- |
| **Table S1**  The active ingredients of HJJPD | 2-5 |
| **Table S2** The active ingredients of HJJPD in NAFLD | 6-7 |
| **Table S3** The target of top 10 proteins | 8 |
| **Table S4 and S5** The raw data of manuscript drawing | 9-12 |

**Table S1 The active ingredients of HJJPD**

| **Herbal** | | **Active ingredients** |
| --- | --- | --- |
| Baizhu | | [Trigonelline](http://www.tcmip.cn/TCMIP/index.php/Home/Index/cf_details.html?id=84) |
|  |  | [Fructose](http://www.tcmip.cn/TCMIP/index.php/Home/Index/cf_details.html?id=444) |
|  |  | [Atractylenolide III](http://www.tcmip.cn/TCMIP/index.php/Home/Index/cf_details.html?id=532) |
|  |  | [6-Methoxy-7-Hydroxycoumarin](http://www.tcmip.cn/TCMIP/index.php/Home/Index/cf_details.html?id=536) |
|  |  | [Atractylenolide I](http://www.tcmip.cn/TCMIP/index.php/Home/Index/cf_details.html?id=537) |
|  |  | 8β-Ethoxy atractylenolide Ⅲ |
|  |  | [(+)-Eudesma-4(15),7(11)-Dien-8-One](http://www.tcmip.cn/TCMIP/index.php/Home/Index/cf_details.html?id=541) |
|  |  | [Juniper Camphor](http://www.tcmip.cn/TCMIP/index.php/Home/Index/cf_details.html?id=542) |
| Huangqi | [Choline](http://www.tcmip.cn/TCMIP/index.php/Home/Index/cf_details.html?id=776) | |
|  | [Kumatakenin](http://www.tcmip.cn/TCMIP/index.php/Home/Index/cf_details.html?id=2436) | |
|  | [Glucuronic Acid](http://www.tcmip.cn/TCMIP/index.php/Home/Index/cf_details.html?id=3140) | |
|  | [Astragaloside I](http://www.tcmip.cn/TCMIP/index.php/Home/Index/cf_details.html?id=3128) | |
|  | [Astragaloside Ii](http://www.tcmip.cn/TCMIP/index.php/Home/Index/cf_details.html?id=3129) | |
|  | [Astragaloside Iii](http://www.tcmip.cn/TCMIP/index.php/Home/Index/cf_details.html?id=3130) | |
|  | [Astragaloside Iv](http://www.tcmip.cn/TCMIP/index.php/Home/Index/cf_details.html?id=3131) | |
|  | [Astragaloside V](http://www.tcmip.cn/TCMIP/index.php/Home/Index/cf_details.html?id=3132) | |
|  | [Astragaloside VI](http://www.tcmip.cn/TCMIP/index.php/Home/Index/cf_details.html?id=3133) | |
|  | [Astragaloside VI](http://www.tcmip.cn/TCMIP/index.php/Home/Index/cf_details.html?id=3134)I | |
|  | [Astragaloside VI](http://www.tcmip.cn/TCMIP/index.php/Home/Index/cf_details.html?id=3135)II | |
|  | [2'-Hydroxy-3',4'-Dimethoxy-Isoflavane-7-O-β-D-Glucoside](http://www.tcmip.cn/TCMIP/index.php/Home/Index/cf_details.html?id=3141) | |
| Baxia | [Shogaol](http://www.tcmip.cn/TCMIP/index.php/Home/Index/cf_details.html?id=767) | |
|  | [Baicalin](http://www.tcmip.cn/TCMIP/index.php/Home/Index/cf_details.html?id=770) | |
|  | [Baicalein](http://www.tcmip.cn/TCMIP/index.php/Home/Index/cf_details.html?id=771) | |
|  | [Coniine](http://www.tcmip.cn/TCMIP/index.php/Home/Index/cf_details.html?id=777) | |
|  | [Ephedrine](http://www.tcmip.cn/TCMIP/index.php/Home/Index/cf_details.html?id=778) | |
|  | [Salicylic Acid](http://www.tcmip.cn/TCMIP/index.php/Home/Index/cf_details.html?id=218) | |
| Chenpi | [3-O-trans ferulylquinic acid](http://www.tcmip.cn/TCMIP/index.php/Home/Index/cf_details.html?id=17) | |
|  | [5-Hydroxy-6,7,3',4'-Tetramethoxyflavone](http://www.tcmip.cn/TCMIP/index.php/Home/Index/cf_details.html?id=166) | |
|  | [Rosmarinic Acid](http://www.tcmip.cn/TCMIP/index.php/Home/Index/cf_details.html?id=814) | |
|  | [Citronellol](http://www.tcmip.cn/TCMIP/index.php/Home/Index/cf_details.html?id=817) | |
|  | [Geraniol](http://www.tcmip.cn/TCMIP/index.php/Home/Index/cf_details.html?id=850) | |
|  | [Nobiletin](http://www.tcmip.cn/TCMIP/index.php/Home/Index/cf_details.html?id=1348) | |
|  | [Tangeretin](http://www.tcmip.cn/TCMIP/index.php/Home/Index/cf_details.html?id=1349) | |
|  | [5,5'-Oxydimethylene-Bis(2-Furaldehyde)](http://www.tcmip.cn/TCMIP/index.php/Home/Index/cf_details.html?id=1362) | |
|  | [5-Hydroxy-7,8,4'-Trimethoxyflavanone](http://www.tcmip.cn/TCMIP/index.php/Home/Index/cf_details.html?id=1363) | |
|  | [Neohesperidin](http://www.tcmip.cn/TCMIP/index.php/Home/Index/cf_details.html?id=1369) | |
|  | [Skullcapflavone II](http://www.tcmip.cn/TCMIP/index.php/Home/Index/cf_details.html?id=1366) | |
| Fuling | [Lauric Acid](http://www.tcmip.cn/TCMIP/index.php/Home/Index/cf_details.html?id=260) | |
|  | [25-Hydroxy-3-Epidehydrotumulosic Acid](http://www.tcmip.cn/TCMIP/index.php/Home/Index/cf_details.html?id=2268) | |
|  | [Poricoic Acid A](http://www.tcmip.cn/TCMIP/index.php/Home/Index/cf_details.html?id=2274) | |
|  | [Poricoic Acid B](http://www.tcmip.cn/TCMIP/index.php/Home/Index/cf_details.html?id=2275) | |
|  | [Poricoic Acid C](http://www.tcmip.cn/TCMIP/index.php/Home/Index/cf_details.html?id=2276) | |
|  | [Poricoic Acid D](http://www.tcmip.cn/TCMIP/index.php/Home/Index/cf_details.html?id=2277) | |
| Cangzhu | [Epigallocatechin](http://www.tcmip.cn/TCMIP/index.php/Home/Index/cf_details.html?id=352) | |
|  | [(+)-Eudesma-4(15),7(11)-Dien-8-One](http://www.tcmip.cn/TCMIP/index.php/Home/Index/cf_details.html?id=541) | |
|  | [Atractyloside A](http://www.tcmip.cn/TCMIP/index.php/Home/Index/cf_details.html?id=1046) | |
|  | [Atractyloside A 14-O-Î’-D-Fructofuranoside](http://www.tcmip.cn/TCMIP/index.php/Home/Index/cf_details.html?id=1047) | |
|  | [Atractyloside C](http://www.tcmip.cn/TCMIP/index.php/Home/Index/cf_details.html?id=1049) | |
|  | [Atractyloside G](http://www.tcmip.cn/TCMIP/index.php/Home/Index/cf_details.html?id=1052) | |
|  | [(2E,8E)-2,8-Decadiene-4,6-Diyne-1,10-Diol 1-O-β-D-Glucopyranoside](http://www.tcmip.cn/TCMIP/index.php/Home/Index/cf_details.html?id=1057) | |
|  | [10-Epiatractyloside A](http://www.tcmip.cn/TCMIP/index.php/Home/Index/cf_details.html?id=1059) | |
|  | [3Î’-Hydroxyatractylone](http://www.tcmip.cn/TCMIP/index.php/Home/Index/cf_details.html?id=1064) | |
|  | [(5R,7R,10S)-Isopterocarpolon-β-D-Glucopyranoside](http://www.tcmip.cn/TCMIP/index.php/Home/Index/cf_details.html?id=1065) | |
|  | [(1S,5R,7R,10R)-Secoatractylolactone 11-O-Î’-D-Glucopyranoside](http://www.tcmip.cn/TCMIP/index.php/Home/Index/cf_details.html?id=1069) | |
|  | Atractylodin | |
|  | [Syringin](http://www.tcmip.cn/TCMIP/index.php/Home/Index/cf_details.html?id=1070) | |
| Zhishi | [Pavilion](http://www.tcmip.cn/TCMIP/index.php/Home/Index/cf_details.html?id=84) | |
|  | [Nobiletin](http://www.tcmip.cn/TCMIP/index.php/Home/Index/cf_details.html?id=1348) | |
|  | [Tangeretin](http://www.tcmip.cn/TCMIP/index.php/Home/Index/cf_details.html?id=1349) | |
|  | [Coniferin](http://www.tcmip.cn/TCMIP/index.php/Home/Index/cf_details.html?id=1546) | |
|  | [Naringenin](http://www.tcmip.cn/TCMIP/index.php/Home/Index/cf_details.html?id=2962) | |
|  | [Sinensetin](http://www.tcmip.cn/TCMIP/index.php/Home/Index/cf_details.html?id=3469) | |
|  | [Isosinensetin](http://www.tcmip.cn/TCMIP/index.php/Home/Index/cf_details.html?id=4384) | |
|  | [Synephrine](http://www.tcmip.cn/TCMIP/index.php/Home/Index/cf_details.html?id=5376) | |
|  | [5,7,8,4'-Tetramethoxyflavone](http://www.tcmip.cn/TCMIP/index.php/Home/Index/cf_details.html?id=6189) | |
|  | [5,7-Dihydroxycoumarin](http://www.tcmip.cn/TCMIP/index.php/Home/Index/cf_details.html?id=6405) | |
|  | [Hesperitin](http://www.tcmip.cn/TCMIP/index.php/Home/Index/cf_details.html?id=6406) | |
| Zexie | [Alisol A](http://www.tcmip.cn/TCMIP/index.php/Home/Index/cf_details.html?id=6054) | |
|  | [Alisol B](http://www.tcmip.cn/TCMIP/index.php/Home/Index/cf_details.html?id=6056) | |
|  | [Alisol C](http://www.tcmip.cn/TCMIP/index.php/Home/Index/cf_details.html?id=6058) | |
|  | [Neoalisol](http://www.tcmip.cn/TCMIP/index.php/Home/Index/cf_details.html?id=6069) | |
|  | [Oriediterpenol](http://www.tcmip.cn/TCMIP/index.php/Home/Index/cf_details.html?id=6070) | |
|  | [Oriediterpenoside](http://www.tcmip.cn/TCMIP/index.php/Home/Index/cf_details.html?id=6071) | |
|  | [Alisol A Monoacetate](http://www.tcmip.cn/TCMIP/index.php/Home/Index/cf_details.html?id=6055) | |
|  | [Alisol B Monoacetate](http://www.tcmip.cn/TCMIP/index.php/Home/Index/cf_details.html?id=6057) | |
|  | [Alisol C Monoacetate](http://www.tcmip.cn/TCMIP/index.php/Home/Index/cf_details.html?id=6059) | |
|  | [Alisol E 23-Acetate](http://www.tcmip.cn/TCMIP/index.php/Home/Index/cf_details.html?id=6060) | |
|  | [Alisol E 24-Acetate](http://www.tcmip.cn/TCMIP/index.php/Home/Index/cf_details.html?id=6061) | |
|  | [Alizexol A](http://www.tcmip.cn/TCMIP/index.php/Home/Index/cf_details.html?id=6062) | |
|  | [Alizexol B](http://www.tcmip.cn/TCMIP/index.php/Home/Index/cf_details.html?id=6063) | |
| Chuangxiong | [3-O-trans ferulylquinic acid](http://www.tcmip.cn/TCMIP/index.php/Home/Index/cf_details.html?id=17) | |
|  | [Caffeic Acid](http://www.tcmip.cn/TCMIP/index.php/Home/Index/cf_details.html?id=97) | |
|  | [Protocatechuic Acid](http://www.tcmip.cn/TCMIP/index.php/Home/Index/cf_details.html?id=406) | |
|  | [Chrysophanic Acid](http://www.tcmip.cn/TCMIP/index.php/Home/Index/cf_details.html?id=1434) | |
|  | [3-N-Butyl-3-Hydroxy-4,5,6,7-Tetrahydro-6,7-Dihydroxy Phthalide](http://www.tcmip.cn/TCMIP/index.php/Home/Index/cf_details.html?id=1445) | |
|  | [Chrysophanol](http://www.tcmip.cn/TCMIP/index.php/Home/Index/cf_details.html?id=1449) | |
|  | [(Z)-4,5-Dihydro-6,7-Trans-Dihydroxy-3-Butylidene Phthalide](http://www.tcmip.cn/TCMIP/index.php/Home/Index/cf_details.html?id=1454) | |
|  | [Senkyunolide G](http://www.tcmip.cn/TCMIP/index.php/Home/Index/cf_details.html?id=1481) | |
|  | [Senkyunolide H](http://www.tcmip.cn/TCMIP/index.php/Home/Index/cf_details.html?id=1482) | |
|  | [Senkyunolide J](http://www.tcmip.cn/TCMIP/index.php/Home/Index/cf_details.html?id=1483) | |
|  | [Senkyunolide N](http://www.tcmip.cn/TCMIP/index.php/Home/Index/cf_details.html?id=1487) | |
|  | [Wallichilide](http://www.tcmip.cn/TCMIP/index.php/Home/Index/cf_details.html?id=1492) | |
| Heye | [Nuciferine](http://www.tcmip.cn/TCMIP/index.php/Home/Index/cf_details.html?id=1774) | |
|  | [Armepavine](http://www.tcmip.cn/TCMIP/index.php/Home/Index/cf_details.html?id=2775) | |
|  | [Lirinidine](http://www.tcmip.cn/TCMIP/index.php/Home/Index/cf_details.html?id=2777) | |
|  | [N-Methylasimilobine](http://www.tcmip.cn/TCMIP/index.php/Home/Index/cf_details.html?id=2778) | |
|  | [D-N-Methyl Coclaurine](http://www.tcmip.cn/TCMIP/index.php/Home/Index/cf_details.html?id=2779) | |
|  | [Pronuciferine](http://www.tcmip.cn/TCMIP/index.php/Home/Index/cf_details.html?id=2783) | |
| Yiyiren | Monoolein | |
|  | 2-Monoolein | |

**Table S2 The active ingredients of HJJPD in NAFLD**

| **Herbal** | **The active ingredients of HJJPD** |
| --- | --- |
| Baizhu | [Trigonelline](http://www.tcmip.cn/TCMIP/index.php/Home/Index/cf_details.html?id=84) |
| Baizhu | [Fructose](http://www.tcmip.cn/TCMIP/index.php/Home/Index/cf_details.html?id=444) |
| Baizhu | [Atractylenolide III](http://www.tcmip.cn/TCMIP/index.php/Home/Index/cf_details.html?id=532) |
| Baizhu | [6-Methoxy-18-Hydroxycoumarin](http://www.tcmip.cn/TCMIP/index.php/Home/Index/cf_details.html?id=536) |
| Baizhu | -Eudesma-4,7-Dien-11-One |
| Baizhu | [Juniper Camphor](http://www.tcmip.cn/TCMIP/index.php/Home/Index/cf_details.html?id=542) |
| Banxia | [Baicalin](http://www.tcmip.cn/TCMIP/index.php/Home/Index/cf_details.html?id=770) |
| Banxia | [Ephedrine](http://www.tcmip.cn/TCMIP/index.php/Home/Index/cf_details.html?id=778) |
| Banxia | [Baicalein](http://www.tcmip.cn/TCMIP/index.php/Home/Index/cf_details.html?id=771) |
| Banxia | Salicylic Acid |
| Cangzhu | Epigallocatechin |
| Cangzhu | [(5R,7R,17S)-IsopterocarpolonÎ’-D-Glucopyranoside](http://www.tcmip.cn/TCMIP/index.php/Home/Index/cf_details.html?id=1065) |
| Cangzhu | (+)-Eudesma-4(15),7(11)-Dien-11-One |
| Cangzhu | AtractylosideA17-O-Î’-D-Fructofuranoside |
| Cangzhu | [Atractyloside C](http://www.tcmip.cn/TCMIP/index.php/Home/Index/cf_details.html?id=1049) |
| Cangzhu | [Atractyloside G](http://www.tcmip.cn/TCMIP/index.php/Home/Index/cf_details.html?id=1052) |
| Cangzhu | [6Î’-Hydroxyatractylone](http://www.tcmip.cn/TCMIP/index.php/Home/Index/cf_details.html?id=1064) |
| Chenpi | [3-O-transferulylquinic acid](http://www.tcmip.cn/TCMIP/index.php/Home/Index/cf_details.html?id=17) |
| Chenpi | [Neohesperidin](http://www.tcmip.cn/TCMIP/index.php/Home/Index/cf_details.html?id=1369) |
| Chenpi | [Skullcap flavone II](http://www.tcmip.cn/TCMIP/index.php/Home/Index/cf_details.html?id=1366) |
| Chenpi | [5-Hydroxy-6,7,3',13'-Tetramethoxyflavone](http://www.tcmip.cn/TCMIP/index.php/Home/Index/cf_details.html?id=166) |
| Chenpi | [5,5'-Oxydimethylene-Bis(2-Furaldehyde)](http://www.tcmip.cn/TCMIP/index.php/Home/Index/cf_details.html?id=1362) |
| Chuanxiong | [3-O-transferulylquinic acid](http://www.tcmip.cn/TCMIP/index.php/Home/Index/cf_details.html?id=17) |
| Chuanxiong | [Wallichilide](http://www.tcmip.cn/TCMIP/index.php/Home/Index/cf_details.html?id=1492) |
| Chuanxiong | [Caffeic Acid](http://www.tcmip.cn/TCMIP/index.php/Home/Index/cf_details.html?id=97) |
| Chuanxiong | [Protocatechuic Acid](http://www.tcmip.cn/TCMIP/index.php/Home/Index/cf_details.html?id=406) |
| Chuanxiong | [Chrysophanic Acid](http://www.tcmip.cn/TCMIP/index.php/Home/Index/cf_details.html?id=1434) |
| Chuanxiong | [Chrysophanol](http://www.tcmip.cn/TCMIP/index.php/Home/Index/cf_details.html?id=1449) |
| Fuling | [Lauric Acid](http://www.tcmip.cn/TCMIP/index.php/Home/Index/cf_details.html?id=260) |
| Fuling | [25-Hydroxy-22-Epidehydrotumulosic Acid](http://www.tcmip.cn/TCMIP/index.php/Home/Index/cf_details.html?id=2268) |
| Fuling | [Poricoic Acid A](http://www.tcmip.cn/TCMIP/index.php/Home/Index/cf_details.html?id=2274) |
| Fuling | [Poricoic Acid B](http://www.tcmip.cn/TCMIP/index.php/Home/Index/cf_details.html?id=2275) |
| Fuling | [Poricoic Acid D](http://www.tcmip.cn/TCMIP/index.php/Home/Index/cf_details.html?id=2277) |
| Heye | [Nuciferine](http://www.tcmip.cn/TCMIP/index.php/Home/Index/cf_details.html?id=1774) |
| Heye | [Lirinidine](http://www.tcmip.cn/TCMIP/index.php/Home/Index/cf_details.html?id=2777) |
| Heye | [N-Methylasimilobine](http://www.tcmip.cn/TCMIP/index.php/Home/Index/cf_details.html?id=2778) |
| Heye | [Pronuciferine](http://www.tcmip.cn/TCMIP/index.php/Home/Index/cf_details.html?id=2783) |
| Huangqi | [Kumatakenin](http://www.tcmip.cn/TCMIP/index.php/Home/Index/cf_details.html?id=2436) |
| Huangqi | [Astragaloside Ⅷ](http://www.tcmip.cn/TCMIP/index.php/Home/Index/cf_details.html?id=3135) |
| Huangqi | [2'-Hydroxy-3',4'-Dimethoxy-Isoflavane-9-O-Î’-D-Glucoside](http://www.tcmip.cn/TCMIP/index.php/Home/Index/cf_details.html?id=3141) |
| Yiyiren | Monoolein |
| Yiyiren | 4-Monoolein |
| ZeXie | [Alisol A](http://www.tcmip.cn/TCMIP/index.php/Home/Index/cf_details.html?id=6054) |
| ZeXie | [Alisol E23-Acetate](http://www.tcmip.cn/TCMIP/index.php/Home/Index/cf_details.html?id=6060) |
| ZeXie | [Alisol E 24-Acetate](http://www.tcmip.cn/TCMIP/index.php/Home/Index/cf_details.html?id=6061) |
| ZeXie | [Alizexol A](http://www.tcmip.cn/TCMIP/index.php/Home/Index/cf_details.html?id=6062) |
| ZeXie | [Alizexol B](http://www.tcmip.cn/TCMIP/index.php/Home/Index/cf_details.html?id=6063) |
| ZeXie | [Alisol B](http://www.tcmip.cn/TCMIP/index.php/Home/Index/cf_details.html?id=6056) |
| ZeXie | [Alisol C](http://www.tcmip.cn/TCMIP/index.php/Home/Index/cf_details.html?id=6058) |
| ZeXie | [Neoalisol](http://www.tcmip.cn/TCMIP/index.php/Home/Index/cf_details.html?id=6069) |
| ZeXie | [Oriediterpenol](http://www.tcmip.cn/TCMIP/index.php/Home/Index/cf_details.html?id=6070) |
| ZeXie | [Oriediterpenoside](http://www.tcmip.cn/TCMIP/index.php/Home/Index/cf_details.html?id=6071) |
| ZeXie | [Alisol A Monoacetate](http://www.tcmip.cn/TCMIP/index.php/Home/Index/cf_details.html?id=6055) |
| ZeXie | [Alisol B Monoacetate](http://www.tcmip.cn/TCMIP/index.php/Home/Index/cf_details.html?id=6057) |
| ZeXie | [Alisol C Monoacetate](http://www.tcmip.cn/TCMIP/index.php/Home/Index/cf_details.html?id=6059) |

**Table S3** The target of top 10 proteins

| Target | PDB ID |
| --- | --- |
| TNF | 2E7A |
| IL6 | [4O9H](https://www.rcsb.org/structure/4O9H) |
| AKT1 | 4EJN |
| IL1B | 6Y8I |
| PPARG | 2VST |
| PTGS2 | 5IKV |
| ESR1 | 4XI3 |
| LPL | 6OB0 |
| PPARA | 3FEI |
| HMGCR | 1HWI |

**Table S4** The raw data of manuscript drawing

|  | Normal | Model | Orlistat | Low dose | Medium dose | High dose |
| --- | --- | --- | --- | --- | --- | --- |
| Live weight | 1.36 | 2.18 | 1.63 | 1.81 | 1.68 | 1.5 |
|  | 1.54 | 2.33 | 1.43 | 1.68 | 1.66 | 1.43 |
|  | 1.43 | 2.13 | 1.73 | 1.61 | 1.9 | 1.39 |
|  | 1.52 | 2.06 | 1.56 | 1.87 | 1.65 | 1.68 |
|  | 1.68 | 2.21 | 1.67 | 1.8 | 1.64 | 1.7 |
|  | 1.41 | 2.33 | 1.49 | 1.63 | 1.78 | 1.53 |
|  | 1.38 | 2.14 | 1.39 | 1.89 | 1.97 | 1.7 |
|  | 1.68 | 2.07 | 1.47 | 1.96 | 1.86 | 1.83 |
| Live/body weight ratio | 5.33 | 4.41 | 3.68 | 4.01 | 3.78 | 3.55 |
|  | 5.36 | 4.43 | 3.87 | 4 | 3.71 | 3.52 |
|  | 5.63 | 4.32 | 3.82 | 4.19 | 3.63 | 3.49 |
|  | 5.23 | 4.31 | 3.65 | 4.12 | 3.84 | 3.41 |
|  | 5.35 | 4.5 | 3.51 | 3.99 | 3.9 | 3.66 |
|  | 4.99 | 4.66 | 3.49 | 3.86 | 3.85 | 3.68 |
|  | 5.54 | 4.22 | 3.59 | 4.02 | 3.72 | 3.58 |
|  | 4.96 | 4.52 | 3.69 | 3.78 | 3.84 | 3.41 |
|  | 5.65 | 4.39 | 3.84 | 4.12 | 3.76 | 3.64 |
| AST | 81.32 | 125.64 | 107.36 | 122.48 | 120.17 | 118.36 |
|  | 81.45 | 117.53 | 110.42 | 125.69 | 117.34 | 106.46 |
|  | 79.34 | 128.08 | 104.35 | 113.16 | 105.43 | 117.74 |
|  | 78.95 | 125.67 | 106.07 | 117.88 | 113.91 | 108.19 |
|  | 83.11 | 120.19 | 111.82 | 120.42 | 106.68 | 118.04 |
|  | 82.41 | 128.37 | 114.09 | 118.36 | 104.13 | 112.85 |
|  | 78.45 | 122.69 | 113.25 | 116.97 | 114.88 | 109.79 |
|  | 80.12 | 118.85 | 107.48 | 118.03 | 110.02 | 115.58 |
| ALT | 25.36 | 74.35 | 35.76 | 48.25 | 43.24 | 30.37 |
|  | 27.08 | 73.61 | 36.49 | 50.31 | 39.98 | 27.61 |
|  | 22.19 | 72.19 | 33.08 | 47.28 | 42.26 | 29.99 |
|  | 21.39 | 69.08 | 32.35 | 46.65 | 37.39 | 33.32 |
|  | 24.76 | 68.33 | 34.14 | 51.09 | 39.45 | 30.25 |
|  | 26.94 | 75.51 | 37.05 | 46.72 | 42.56 | 29.08 |
|  | 25.33 | 74.08 | 33.5 | 50.39 | 39.81 | 34.07 |
|  | 24.9 | 73.22 | 34.85 | 49.6 | 40.04 | 28.12 |
| TC | 2.38 | 4.01 | 3.75 | 4.11 | 3.91 | 3.06 |
|  | 2.4 | 3.76 | 3.63 | 3.94 | 3.65 | 2.92 |
|  | 2.21 | 4.08 | 3.29 | 3.62 | 3.57 | 3.39 |
|  | 2.03 | 3.59 | 3.67 | 3.85 | 3.77 | 3.12 |
|  | 2.25 | 4.28 | 3.48 | 3.39 | 3.41 | 3.27 |
|  | 2.54 | 3.49 | 3.34 | 3.46 | 3.69 | 3.29 |
|  | 2.16 | 3.68 | 3.43 | 3.82 | 3.38 | 3.08 |
|  | 2.08 | 4.17 | 3.27 | 3.9 | 3.93 | 2.94 |
| TG | 0.73 | 1.34 | 0.91 | 0.83 | 0.86 | 0.75 |
|  | 0.65 | 1.15 | 0.84 | 0.95 | 0.89 | 0.82 |
|  | 0.82 | 1.29 | 0.87 | 1.06 | 0.72 | 0.66 |
|  | 0.71 | 1.33 | 0.96 | 0.85 | 0.85 | 0.89 |
|  | 0.69 | 1.26 | 1.05 | 0.93 | 0.83 | 0.73 |
|  | 0.75 | 1.13 | 0.94 | 0.79 | 0.94 | 0.78 |
|  | 0.82 | 1.18 | 0.86 | 0.96 | 0.72 | 0.81 |
|  | 0.75 | 1.3 | 0.89 | 0.92 | 0.85 | 0.71 |
| HDL | 1.43 | 2.47 | 2.19 | 2.43 | 2.23 | 1.98 |
|  | 1.61 | 2.38 | 2.24 | 2.15 | 1.89 | 1.91 |
|  | 1.59 | 2.35 | 2.07 | 2.26 | 2.05 | 2.04 |
|  | 1.62 | 2.62 | 2.15 | 2.07 | 2.11 | 1.99 |
|  | 1.41 | 2.56 | 2.17 | 2.51 | 2.25 | 2.17 |
|  | 1.23 | 2.41 | 2.18 | 2.14 | 2.18 | 2.11 |
|  | 1.29 | 2.33 | 2.13 | 2.08 | 2.09 | 2.03 |
|  | 1.34 | 2.6 | 2.21 | 2.32 | 2.21 | 2.01 |
| LDL | 0.36 | 1.61 | 0.8 | 0.87 | 0.8 | 0.75 |
|  | 0.39 | 1.63 | 0.71 | 0.78 | 0.83 | 0.67 |
|  | 0.32 | 1.67 | 0.74 | 0.96 | 0.81 | 0.62 |
|  | 0.35 | 1.52 | 0.64 | 0.86 | 0.92 | 0.71 |
|  | 0.29 | 1.47 | 0.75 | 0.93 | 0.88 | 0.75 |
|  | 0.34 | 1.43 | 0.77 | 0.82 | 0.76 | 0.64 |
|  | 0.31 | 1.54 | 0.8 | 0.83 | 0.98 | 0.66 |
|  | 0.32 | 1.43 | 0.73 | 0.84 | 0.93 | 0.68 |
| AKT1 mRNA levels | 1.38 | 32.65 | 14.39 | 15.97 | 9.03 | 5.41 |
|  | 1.12 | 33.59 | 13.59 | 14.01 | 8.13 | 6.68 |
|  | 1.54 | 34.26 | 12.94 | 16.39 | 9.99 | 4.45 |
|  | 1.98 | 31.9 | 15.37 | 17.76 | 7.3 | 7.13 |
|  | 1.28 | 32.47 | 11.22 | 18.53 | 8.74 | 6.03 |
|  | 1.16 | 29.88 | 14.01 | 16.07 | 10.38 | 5.33 |
|  | 1.67 | 35.45 | 15.08 | 17.26 | 8.48 | 5.78 |
|  | 1.95 | 30.65 | 13.38 | 15.57 | 11.51 | 4.63 |
| PPARG mRNA levels | 1.1 | 6.18 | 2.32 | 3.31 | 2.46 | 1.28 |
|  | 1.15 | 6.02 | 2.65 | 3.25 | 2.71 | 1.66 |
|  | 1.04 | 6.92 | 2.71 | 3.45 | 2.16 | 1.59 |
|  | 0.96 | 6.68 | 2.28 | 3.34 | 2.34 | 1.89 |
|  | 1.13 | 6.34 | 2.36 | 3.67 | 2.48 | 1.57 |
|  | 1.24 | 5.73 | 2.44 | 2.97 | 2.29 | 1.73 |
|  | 1.22 | 5.54 | 2.71 | 2.84 | 2.51 | 1.75 |
|  | 1.08 | 6.01 | 2.6 | 3.69 | 2.56 | 1.68 |
| TNF-α mRNA levels | 1.01 | 8.33 | 2.12 | 6.28 | 2.47 | 1.53 |
|  | 1.05 | 8.61 | 2.05 | 6.34 | 2.44 | 1.66 |
|  | 1.04 | 8.76 | 2.21 | 6.25 | 2.62 | 1.75 |
|  | 1.09 | 8.52 | 2.08 | 6.21 | 2.88 | 1.69 |
|  | 1.1 | 8.65 | 2.16 | 6.19 | 2.91 | 1.67 |
|  | 0.99 | 8.53 | 2.24 | 6.17 | 2.85 | 1.42 |
|  | 1.02 | 8.54 | 2.23 | 6.22 | 2.91 | 1.45 |
|  | 1.08 | 8.61 | 2.25 | 6.49 | 2.76 | 1.48 |
| PTGS2 mRNA levels | 1.08 | 5.82 | 1.85 | 3.75 | 2.76 | 1.33 |
|  | 1.09 | 6.03 | 1.95 | 3.33 | 2.65 | 1.21 |
|  | 1.32 | 5.79 | 2.03 | 3.04 | 2.8 | 1.47 |
|  | 0.97 | 5.93 | 1.56 | 3.04 | 2.47 | 1.18 |
|  | 1.26 | 6.04 | 1.93 | 2.92 | 2.81 | 1.06 |
|  | 1.14 | 5.924 | 1.88 | 3.47 | 2.94 | 1.38 |
|  | 1.3 | 5.48 | 1.69 | 3.82 | 2.84 | 1.24 |
|  | 1.05 | 5.61 | 1.74 | 3.18 | 2.49 | 1.16 |

**Table S5** The raw data of manuscript drawing

| Time  /week | Normal | SD | Model | SD | Orlistat | SD | Low dose | SD | Medium dose | SD | High dose | SD |
| --- | --- | --- | --- | --- | --- | --- | --- | --- | --- | --- | --- | --- |
| 0 | 20.64 | 0.4 | 20.38 | 0.3 | 20.17 | 0.23 | 20.6 | 0.19 | 20.14 | 0.15 | 20.45 | 0.16 |
| 4 | 23.32 | 0.3 | 28.57 | 0.4 | 26.36 | 0.18 | 26.08 | 0.24 | 27.41 | 0.27 | 26.36 | 0.25 |
| 8 | 24.84 | 0.6 | 35.34 | 0.33 | 31.54 | 0.26 | 31.32 | 0.22 | 34.6 | 0.21 | 30.93 | 0.32 |
| 12 | 26.03 | 0.3 | 41.24 | 0.52 | 35.09 | 0.31 | 36.43 | 0.37 | 39.82 | 0.38 | 35.35 | 0.29 |
| 16 | 27.51 | 0.3 | 46.21 | 0.24 | 39.25 | 0.25 | 41.33 | 0.34 | 43.01 | 0.32 | 41.25 | 0.37 |
| 20 | 28.04 | 0.5 | 48.44 | 0.28 | 41.12 | 0.54 | 43.56 | 0.46 | 45.21 | 0.47 | 43.12 | 0.34 |
| 24 | 28.14 | 0.4 | 49.43 | 0.41 | 42.087 | 0.49 | 44.51 | 0.40 | 46.75 | 0.29 | 44.91 | 0.39 |
